# Natural Products: Optimizing Cancer Treatment through Modulation of Redox Balance

**DOI:** 10.1155/2020/2407074

**Published:** 2020-06-02

**Authors:** Patrícia Rijo, Milica Pešić, Ana S. Fernandes, Cláudia N. Santos

**Affiliations:** ^1^CBIOS - Universidade Lusófona Research Center for Biosciences & Health Technologies, Lisboa, Portugal; ^2^IMed.ULisboa-Research Institute for Medicines, Lisboa, Portugal; ^3^Institute for Biological Research “Siniša Stanković”-National Institute of Republic of Serbia, University of Belgrade, Belgrade, Serbia; ^4^CEDOC, NOVA Medical School, Faculdade de Ciências Médicas, Universidade NOVA de Lisboa, Lisboa, Portugal

Nature is an inexhaustible reservoir of compounds with healing properties, and people used natural products to treat different medical conditions from ancient times. One of the main features of natural products is their ability to modulate directly and indirectly oxidative stress and protect human cells from aging and death [1]. Besides aging, cardiovascular diseases, chronic obstructive pulmonary disease, chronic kidney disease, and neurodegeneration, cancer can be considered as an oxidative stress-related disease [2]. Therefore, natural products are also valuable for treating cancer. Increased generation of reactive oxygen species (ROS) which leads to oxidative stress and provokes inflammation eventually may cause carcinogenesis or stimulate cancer progression and metastatic behavior [3]. Despite still preserved indigenous knowledge about plants and other organisms with medicinal value, clinical development of natural preparations (both compounds and extracts) is difficult and slow. However, some of the most exploited chemotherapeutics have natural origin such as doxorubicin, vincristine, and paclitaxel [4]. Today, we know that natural compounds may also cause unwanted effects particularly due to their interference with redox balance [5].

Therefore, advanced and innovative studies of natural product interactions with human metabolism are warranted. The diversity of natural products should be the advantage in our search for the best anticancer nature-inspired drugs.

This special issue assembles twelve contributions (three reviews and nine original articles) regarding the anticancer effects of different natural products and their derivatives, particularly those with the potential to modulate oxidative-stress in cancer.

A review article by J. T. de Giffoni de Carvalho et al. explores medicinal plants found in the Brazilian Cerrado tropical savanna ecoregion emphasizing the antioxidant properties of their extracts as well as their potential for cell death induction in different malignant cells. Moreover, the authors comprehensively describe other medicinal plants from the same region which showed protective capacity against chemotherapy-induced cell toxicity.

Another review article by P. Aiello et al. comprehensively investigates medicinal plants in the prevention and treatment of colon cancer. Their study revealed that grape, soybean, green tea, garlic, olive, and pomegranate are the most effective plants against colon cancer. Diverse in vitro and in vivo models provided evidence that fruits, seeds, leaves, and roots of these plants are abundant in saponins, polysaccharides, triterpenoids, alkaloids, polyphenol glycosides, including flavonoids, and simple phenols, such as caffeic acid, catechins, quercetin and luteolin, and kaempferol and luteolin glycosides. These natural compounds exert various effects such as induction of superoxide dismutase, reduction of DNA oxidation, cell cycle arrest in S phase, suppression of prosurvival signaling pathways and cell invasion, reduction of antiapoptotic and increase of proapoptotic factors, and decrease of proliferating cell nuclear antigen (PCNA), cyclin A, cyclin D1, cyclin B1, and cyclin E.

An interesting overview of melatonin protective action against the side effects of chemotherapy is given by Z. Ma et al. Melatonin easily crosses all biological barriers while its concentration within the cells is particularly high in mitochondria. This is important for its ability to resist mitochondrial oxidative-stress damage. In cancer as well as in other aging-related diseases, melatonin can reduce mitochondrial-mediated cell death and thus protect normal cells against the harmful effects of anthracyclines, alkylating agents, platinum compounds, antimetabolites, mitotic inhibitors, and novel targeted therapies.

The remaining original articles are focused on already isolated and identified natural compounds or plant extracts tested for their anticancer effects and protective effects against classic chemotherapy in different cancer models.

D. T. H. Castro et al. investigated antimelanoma effects of ethanolic extract of *Senna velutina* roots shown to contain flavonoid derivatives of the catechin, anthraquinone, and piceatannol groups. B16F10-Nex2 murine cell line was used for the assessment of extract's effects. Results showed that the extract induced apoptotic cell death followed by caspase-3 activation and increased intracellular calcium and ROS levels. Tumor volume and pulmonary metastasis were followed after subcutaneous implantation of B16F10-Nex2 cells in the lumbosacral region of C57Bl/6 mice. Importantly, it was shown that both the primary tumor volume and the number of pulmonary nodules decreased over 50% when this ethanolic extract was applied.

H. P. Vasantha Rupasinghe et al. showed that the application of a bioconversion process using probiotic bacteria *Lactobacillus rhamnosus* can enhance the pharmacological activities of cranberry extracts probably by generating more active metabolites. The proanthocyanidin-rich extract exposed to the bacteria was particularly active against HepG2 cells inducing significantly stronger mitochondria-dependent apoptosis when compared to parental extract which was not exposed to probiotic bacteria.

M. Shen et al. showed both in vitro and in vivo that betulinic acid induces ROS-dependent apoptosis by inhibiting the NF-*κ*B pathway in human multiple myeloma. Betulinic acid exerted its effect mainly through mitochondrial apoptosis induction, cell cycle blockade, mitochondrial membrane potential disruption, intracellular ROS accumulation, and NF-*κ*B signaling inhibition. This comprehensive study above all elucidated the complex regulatory interaction between ROS and the NF-*κ*B pathway in multiple myeloma.

E. Hernández-SanMiguel et al. found that Ocoxin® oral solution affects stem cell properties in certain primary glioblastoma cells by inhibiting their self-renewal capacity. Moreover, systemic treatment of animals bearing heterotopic and orthotopic xenografts with Ocoxin® reduced tumor burden. Importantly, Ocoxin® exerted a direct effect on macrophage polarization in vitro and in vivo, inhibiting the protumoral features of M2 macrophages.

T. Kowalczyk et al. studied protective antioxidant and anti-inflammatory properties of aqueous methanolic extracts derived from the aerial parts and roots of in vitro grown *Menyanthes trifoliata* L. plants on human umbilical vein endothelial cells. The authors found that both extracts demonstrated protective effects against mitochondrial and nuclear DNA damage caused by ROS. Due to the higher content of selected phenolic compounds and betulinic acid in the root extract, it exerted stronger effects than the extract from the aerial part.

G. Isani et al. renewed the interest for the traditional Chinese medicinal plant *Artemisia annua* L. from which famous antimalarial drug artemisinin was isolated. The authors showed that both artemisinin and hydroalcoholic plant extract induced a cytotoxic effect in D-17 canine osteosarcoma cells. Pure artemisinin caused an increase of cells in the S phase, whereas the hydroalcoholic extract induced G2/M arrest. A significant decrease of iron concentration was also observed indicating that ferroptosis as a specific cell death type might contribute to the artemisinin effect. The hydroalcoholic extract was more potent than pure artemisinin demonstrating a possible synergistic effect of its other components with artemisinin.

D. Pang et al. provided the evidence that polyphyllin VII isolated from *Paris polyphylla* var. yunnanensis induces apoptosis and autophagic cell death via ROS-provoked suppression of AKT/mTORC1 signaling cascade. Moreover, polyphyllin VII in combination with temozolomide showed synergistic interaction followed by a decrease of MGMT expression. Therefore, polyphyllin VII can be considered as a valuable drug to attenuate the ability of glioma cells to repair the temozolomide-induced DNA methylation and reduce the resistance to temozolomide.

I. Kumburovic et al. showed that anxiogenic manifestation of widely used chemotherapeutic cisplatin caused by increased oxidative stress and proapoptotic effect in the hippocampus can be attenuated by supplementation with *Satureja hortensis* L. methanolic extract in rats. This work suggests a beneficial role of these natural products in the protection of cisplatin-induced neurotoxicity.

B. Yang et al. demonstrated that tetramethylpyrazine, an alkaloid extracted from the roots of *Ligusticum chuanxiong* Hort, can overcome doxorubicin-induced endothelian toxicity. To that end, the authors used vascular endothelium injury models in mice and human umbilical vein endothelial cells and showed that tetramethylpyrazine protects the vascular endothelium against doxorubicin-induced injury via upregulating 14-3-3*γ* expression, promoting translocation of Bcl-2 to the mitochondria, closing mitochondria permeability transition pore, maintaining mitochondrial membrane potential, and suppressing oxidative stress.

Overall, the articles presented in this special issue provide experimental evidence and assembled scientific data, which clearly emphasize the medicinal value of natural products to fight cancer and prevent side effects of approved chemotherapeutics by modulating oxidative stress. Our planet has to offer plenty of bioactive molecules of which some are not yet discovered, and this raises the hope that in the future we will be able to sustainably exploit natural resources and find more potent and safer anticancer agents.

*Patrícia Rijo* *Milica Pešić* *Ana S. Fernandes* *Cláudia N. Santos*
